# Supplementary material for: Feasibility and Reliability of the Osteoarthritis Quality Indicator Questionnaire for Assessing Osteoarthritis Care in Bilingual General Practices in South Tyrol/Alto Adige, Italy
Source: Medicina (Kaunas). 2025 Oct 26;61(11):1921. doi: 10.3390/medicina61111921 (PMC12654064; doi:10.3390/medicina61111921)
Supplement: Supplementary file 1 [file medicina-61-01921-s001.zip › medicina-3940644-supplementary.pdf]

Supplementary Materials

Table S1: OA-QI v3 Items in English, German, and Italian.

Item	English (OA-QI v3)	German (OA-QI v3-D)	Italian (OA-QI v3-I)
1	Have you been offered information about osteoarthritis?	Wurde Ihnen Information über Arthrose angeboten?	Le sono state offerte informazioni sull'artrosi?
2	Have you been offered information about treatment options for your osteoarthritis?	Wurden Ihnen Informationen zu den Behandlungsmöglichkeiten Ihrer Arthrose angeboten?	Le sono state offerte informazioni riguardo le opzioni per il trattamento della Sua artrosi?
3	Have you been offered information about how you can manage your osteoarthritis?	Wurden Sie dahingehend beraten, wie Sie selbst mit Ihrer Arthrose umgehen können?	Le sono state offerte informazioni su come può gestire la Sua artrosi?
4	Have you been advised that exercise and physical activity are important to help your osteoarthritis?	Wurden Sie dahingehend beraten, dass Bewegung und körperliche Ertüchtigung wichtig sind, um Ihre Arthrose zu lindern?	È stato/a informato/a dell'importanza dell'esercizio fisico e dell'attività fisica nella gestione della Sua artrosi?
5	Have you been offered guidance on how you can exercise your joints and be physically active?	Wurde Ihnen eine Anleitung angeboten, wie Sie sich bewegen und körperlich aktiv sein können, um Ihre Arthrose zu lindern?	Le sono stati offerti dei consigli su come fare esercizio fisico e mantenersi attivo/a per gestire la Sua artrosi?
6	If you are overweight, have you been advised to try losing weight?	Falls Sie übergewichtig sind: Wurde Ihnen geraten, den Versuch zu unternehmen, Gewicht zu verlieren, um Ihre Arthrose zu lindern?	Nel caso fosse in sovrappeso, Le è stato consigliato di provare a perdere peso per migliorare la Sua artrosi?
7	Have you been offered or given help to lose weight?	Falls Sie übergewichtig sind, wurde Ihnen Hilfe beim Abnehmen angeboten oder bereitgestellt?	Nel caso fosse in sovrappeso, Le è stato offerto o fornito aiuto per perdere peso?
8	Has a health professional discussed with you any problems you may have with daily activities due to your osteoarthritis?	Falls Sie Gelenkschmerzen haben, hat ein/e Arzt/Ärztin oder eine medizinische Fachkraft Sie dazu befragt?	Nel caso avesse dolore articolare, Le sono state chieste informazioni a riguardo da parte di un professionista sanitario?
9	If you have trouble walking, has someone discussed with you if a walking aid like walking sticks, cane, or crutch might be helpful?	Hat ein/e Arzt/Ärztin oder eine medizinische Fachkraft mit Ihnen über mögliche Probleme bei Aktivitäten des täglichen Lebens aufgrund Ihrer Arthrose gesprochen?	Un professionista sanitario ha discusso con Lei eventuali problemi nello svolgimento delle attività quotidiane che siano causati dalla Sua artrosi?
10	If you have trouble working due to your osteoarthritis, have you been offered advice about how to remain in or return to paid or unpaid work?	Falls Sie durch Ihre Arthrose Probleme beim Gehen haben, wurde mit Ihnen besprochen, ob eine Gehhilfe wie Gehstöcke, ein Gehstock oder eine Krücke hilfreich sein könnte?	Nel caso avesse difficoltà a camminare a causa della Sua artrosi, qualcuno ha discusso con Lei se un ausilio come un bastone da passeggio o una stampella potrebbe essere utile?
11	Has a health professional asked you about your joint pain?	Falls Sie durch Ihre Arthrose Probleme bei der Arbeit haben,	Nel caso avesse difficoltà a lavorare a causa della Sua artrosi, Le sono

		wurde Ihnen eine Beratung angeboten, wie Sie in den Beruf zurückkehren oder weiter im Beruf bleiben können?	stati offerti consigli riguardo a come rimanere o tornare al lavoro?
12	Were non-steroidal anti-inflammatory medications the first medication that was recommended to you?	Hat eine medizinische Fachkraft mit Ihnen besprochen, wann Sie zur nächsten Visite oder Kontrolluntersuchung wegen Ihrer Arthrose zurückkehren sollten?	Un professionista sanitario ha discusso con Lei riguardo a quando dovrebbe tornare in visita per la Sua artrosi?
13	If you use non-steroidal anti-inflammatory medication, have you received information about possible side effects?	Falls Sie Gelenkschmerzen oder geschwollene Gelenke haben, wurden Ihnen schmerzstillende entzündungshemmende Medikamente angeboten?	Nel caso avesse dolore o gonfiore articolare, Le sono stati offerti farmaci antidolorifici e antinfiammatori?
14	Has a health professional discussed with you when you should return for another consultation for your osteoarthritis?	Falls Ihnen schmerzstillende entzündungshemmende Medikamente angeboten wurden, wurde Ihnen Information über mögliche Nebenwirkungen angeboten?	Nel caso Le fossero stati offerti farmaci antidolorifici e antinfiammatori, è stato/a informato/a sui possibili effetti collaterali?
15	If you are severely troubled by your osteoarthritis and exercise, medication, or other approaches do not help, have you been offered a referral for an assessment for surgery?	Falls Sie stark unter Arthrose leiden und Bewegung, Medikamente oder andere Behandlungen nicht helfen, wurde Ihnen eine Überweisung zur Beurteilung einer Operation (z. B. Gelenkersatz) angeboten?	Nel caso soffrisse gravemente di artrosi e l'esercizio fisico, i farmaci o altri trattamenti non siano stati d'aiuto, Le è stata offerta una visita per valutare un intervento chirurgico (es. protesi articolare)?
16	If you are severely troubled by pain and other approaches do not help or are unsuitable, have you been offered a steroid injection?	Falls Sie stark unter Gelenkschmerzen leiden und andere Behandlungen nicht helfen oder ungeeignet sind, wurde Ihnen eine Kortison-Injektion angeboten?	Nel caso soffrisse di dolore articolare severo e gli altri trattamenti si fossero rivelati inefficaci o inadatti, Le è stata offerta un'iniezione di cortisone?
17	If you were offered a steroid injection, were you offered information about possible side effects?	Falls Ihnen eine Kortison-Injektion angeboten wurde, wurde Ihnen Information über mögliche Nebenwirkungen angeboten?	Nel caso le fosse stata offerta un'iniezione di cortisone, è stato/a informato/a sui possibili effetti collaterali?

Table S2. Changes Between OA-QI v2 and OA-QI v3.

Item	OA-QI v2 English Text	OA-QI v3 English Text	Type of Change	Rationale
1	Have you been offered information about osteoarthritis?	Same as v2	None	Already clear; unchanged for comparability.
2	Have you been offered information about treatment options for your osteoarthritis?	Same as v2	None	No change required.
3	Have you been offered information about how you can manage your osteoarthritis?	Same as v2	None	Clear and cross-culturally stable.
4	Have you been advised that exercise and physical activity are important to help your osteoarthritis?	Same as v2	None	No change required.
5	Have you been offered guidance on how you can exercise and be physically active to help your osteoarthritis?	Have you been offered guidance on how you can exercise your joints and be physically active?	Minor rewording	Clarifies joint-specific focus.
6	If you are overweight, have you been advised to try losing weight to help your osteoarthritis?	If you are overweight, have you been advised to try losing weight?	Minor rewording	Removes redundancy.
7	If you are overweight, have you been offered or given help to lose weight?	Have you been offered or given help to lose weight?	Scope adjustment	Broadens applicability beyond overweight patients only.
8	Has a health professional discussed with you any problems you may have with daily activities due to your osteoarthritis?	Same as v2	None	No change.
9	If you have trouble walking due to your osteoarthritis, has someone discussed with you if a walking aid like walking sticks, cane, or crutch might be helpful?	If you have trouble walking, has someone discussed with you if a walking aid like walking sticks, cane, or crutch might be helpful?	Minor rewording	Simplifies phrasing; OA context already implicit.
10	If you have trouble working due to your osteoarthritis, have you been offered advice about how to remain in or return to work?	If you have trouble working due to your osteoarthritis, have you been offered advice about how to remain in or return to paid or unpaid work?	Concept expansion	Recognizes unpaid work as relevant to participation.
11	If you have joint pain, has a health professional asked you about your joint pain?	Has a health professional asked you about your joint pain?	Simplification	Removes redundant conditional.
12	If you have joint pain or swollen joints, have you been offered non-steroidal anti-inflammatory medications? (provide examples relevant to the setting/country)	Were non-steroidal anti-inflammatory medications the first medication that was recommended to you?	Substantive revision	Reflects updated OA pharmacologic guidelines emphasizing NSAIDs as first-line therapy.
13	If you were offered non-steroidal anti-inflammatory medications, were you offered information about possible side effects?	If you use non-steroidal anti-inflammatory medication, have you received information about possible side effects?	Minor rewording	Focuses on current users; improves recall accuracy.

14	Has a health professional discussed with you when you should return for another consultation for your osteoarthritis?	Same as v2	None	No change.
15	If you are severely troubled by your osteoarthritis and exercise, medication, or other treatments do not help, have you been offered a referral for an assessment for surgery? (e.g., joint replacement)	If you are severely troubled by your osteoarthritis and exercise, medication, or other approaches do not help, have you been offered a referral for an assessment for surgery?	Minor rewording	Uses broader term “approaches.”
16	If you are severely troubled by joint pain and other treatments do not help or are unsuitable, have you been offered a steroid injection?	If you are severely troubled by pain and other approaches do not help or are unsuitable, have you been offered a steroid injection?	Minor rewording	Streamlines and generalizes terminology.
17	If you were offered a steroid injection, were you offered information about possible side effects?	Same as v2	None	Unchanged.
